# Identifying synergistic combinations of repurposed treatments for Alzheimer’s Disease

**DOI:** 10.1016/j.tjpad.2025.100329

**Published:** 2025-10-27

**Authors:** Clive Ballard, Janet Sultana, Pat Doherty, Gareth Williams, Anne Corbett

**Affiliations:** a College of Medicine & Health, Faculty of Health and Life Sciences, University of Exeter, Exeter EX1 2LU, UK; b Wolfson Sensory, Pain and Regeneration Centre, King’s College London, London SW7 2AZ, UK

**Keywords:** Alzheimer’s, Treatment, Combined, Repurposing

## Abstract

There is considerable opportunity to fast-track novel treatments for Alzheimer’s Disease (AD) through repurposing of existing licensed medications as a way of complementing ongoing drug discovery efforts. Given the complex interplay between AD neuropathological mechanisms, there is also a strong rationale that treatment benefits may be enhanced by examining combinations of treatments to identify potential synergies that would address multiple disease-modifying mechanisms. A Delphi consensus programme combined with a pragmatic analysis of primary care data has identified a series of individual and combined therapies that warrant further investigation in pre-clinical and clinical trials. These include treatments which target well-established neurodegeneration pathways and more explorative agents, including hormonal and anti-infective agents, which align to emerging hypotheses relating to endocrine and immune pathways in AD. Whilst caution is critical when considering combined therapy due to the risks of interaction and polypharmacy, this study provides valuable indications of potential synergistic drug pairs that warrant further investigation.

## 1 Introduction

Alzheimer’s disease (AD) is a devastating neurodegenerative disease that carries a massive personal, financial, and societal impact, with an estimated worldwide cost annually of more than $800 billion [1]. New disease-modifying treatments such as lecanemab and donanemab are emerging from the drug discovery pipeline. For example, lecanemab and donanemab are now licensed in the US and UK [[2], [3], [4], [5]]. Whilst the regulatory approval in some countries is a promising step forward, these treatments still only confer modest benefits [6,7] require complex clinical protocols, and are only likely to be available in selected settings for a small proportion of patients. There is also a promising pipeline of other pharmacological treatments for AD [8]. However, the pathway to approval is lengthy and uncertain. Therefore, in parallel, there is an urgent need for safe and effective compounds that can provide improved therapeutic benefits more broadly to the large numbers of patients with or at risk of developing AD. Our improved understanding of the disease biology has increasingly highlighted several different mechanistic pathways that offer targets for treatment, either alone or in combination, including but not restricted to, tau pathology, neuroinflammation, synaptic function, mitochondrial function, neurogenesis, and neuroprotection [7].

Repurposing existing licensed drugs for other indications as treatments for AD offers a promising and efficient strategy to accelerate the readiness of effective treatments and enrich the treatment pipeline. By identifying medications already approved for other conditions that may have neuroprotective, anti-inflammatory, or cognitive-enhancing effects, repurposing bypasses early-phase safety testing and leverages existing pharmacological data and real-world evidence to significantly reduce time and cost from bench to bedside [9.^10^].

The rationale for investigating combination therapies in AD stems from the growing recognition that the disease is not driven by a single factor but rather by a confluence of interconnected pathological mechanisms [11,12]. Current pharmacological interventions, which largely focus on single molecular targets, have provided only modest and temporary relief from symptoms, without significantly altering the underlying disease trajectory. AD is a multifactorial disorder characterised by the intricate interplay of several key pathological hallmarks, including the extracellular accumulation of amyloid-beta (Aβ) plaques, the intracellular aggregation of neurofibrillary tangles composed of hyperphosphorylated tau protein, chronic neuroinflammation, progressive synaptic dysfunction, cerebrovascular alterations, protein clearance, mitochondrial function, neurochemical and receptor alterations, neurogenesis, neuroprotection, medical risk factors, and lifestyle [13,14]. Although the primary focus of the current paper is to identify potential combination repurposed pharmacological treatments, it should also be acknowledged that approaches combining pharmacological and non-pharmacological interventions may also be valuable. The FINGER study (Finnish Geriatric Intervention Study to Prevent Cognitive Impairment and Disability) is a landmark proof of concept randomized controlled trial designed to assess whether a multi-domain lifestyle intervention could prevent cognitive decline in older adults at risk of dementia. FINGER showed that a combination of diet, exercise, cognitive training and vascular risk management significantly improved cognitive function in older adults at risk of dementia, with excellent long term engagement but a very modest effect size [15]. A further study has recently demonstrated that similar benefits can be achieved with an online multi-domain intervention [16] and similar improvements can also be achieved with online cognitive training [17]. These studies highlight the feasibility and benefit to deliver effective lifestyle interventions, with an excellent opportunity for the future to optimize individual components and improve personalization and to combine these approaches with pharmacological treatments to enhance benefits.

The scientific consensus is increasingly shifting towards the necessity of a paradigm change in AD drug development, moving from a singular target approach to strategies that can simultaneously modulate multiple pathways [18,19]. It therefore makes sense that the most effective treatment approaches will involve a combination of therapies targeting multiple complementary and synergistic processes and pathways as a means of reducing the incidence or progression of AD [20].

### 1.1 Identifying candidate repurposed drugs through a delphi consensus approach

The Delphi expert consensus methodology has been used to identify the best candidates for re-purposing as a program commencing in 2012 [9], with a second iteration in 2020 [10] and a recently completed iteration in 2025[21]. The program involves a panel of 40 global specialists in the clinical and research fields of AD treatment, industry representatives, and a parallel lay panel to advise on candidate acceptability. Members’ identities were anonymised, and feedback was sought on an individual basis, rather than as a group. Panel members suggested drug candidates for initial consideration, including potential symptom– and disease-modifying therapies. The nominations were streamlined to candidates that had been suggested by at least three members, and candidates already being investigated in phase IIb/III trials were excluded. Long-listed candidates were taken forward to systematic review to examine the potential mechanisms, the pre-clinical, clinical, and epidemiological evidence supporting their candidacy. The search used pre-defined queries in MedLine, Cochrane, PsychINFO, and SCOPUS aimed at synthesising evidence concerning the putative mechanism of drug action in AD, the therapeutic effect in vitro and in animal models of AD, evidence of benefit in humans, and safety data. This systematic review was supplemented by additional salient information such as a drug’s blood-brain barrier penetration, license status, posology, and route of administration. Completed reviews were circulated to the panel, and members ranked each candidate based on the strength of evidence, in addition to providing specific comments using a feedback form. Further rounds of feedback and consensus were undertaken as appropriate. Our Delphi methodology and the participating panel members are described in full elsewhere [9,10,21].

In 2012, the priority compounds included glucagon-like peptide 1(GLP-1) analogues, angiotensin receptor blockers (ARBs), calcium antagonists, and tetracycline antibiotics [9]. The outcomes informed clinical trial prioritization and treatment development. Subsequent trials of the ARB losartan [22], the calcium channel blocker nilvadipine [23], and the tetracycline antibiotic minocycline [24] were unsuccessful. However, evidence from national registries, incident AD as a secondary outcome in clinical trials focussing on cardiovascular outcomes [25], and a phase II trial of liraglutide in people with mild-to-moderate AD all indicated benefit [26]. The GLP-1 analogue semaglutide is now moving forward as part of a large Novo-Nordisk phase III clinical trial programme [27,28].

The 2020 Delphi iteration identified the rho-kinase inhibitor fasudil, with impacts on amyloid, tau, and synaptic function, the cholinesterase inhibitor phenserine, with additional impacts on cell death pathways, and anti-viral treatments for herpes simplex as the highest priority candidates [10]. All these compounds are now being investigated in clinical trial programmes.

The most recent Delphi iteration has identified three further high priority candidates [21], the benzothiazole medication riluzole, the vasodilator sildenafil and the herpes zoster vaccine. The evidence supporting these three treatment approaches and the most promising therapies from the two earlier Delphi consensus exercises is summarised in Table 1.

**Table 1:** Summary and supporting evidence for tretment candidates arising from three Delphi consensus programmes.

Drug	Mechanism of action	Pre-clinical data	Clinical data
GLP-1 RA	· Neuroprotection through GSK3β and tau phosphorylation pathways [29,30], · Protection against oxidative stress and apoptotic pathways [31]	· In vitro GLP-1 RA reduces intracellular amyloid precursor protein (APP), Aβ and Fe2+-induced neurodegeneration [29] · In vivo research suggests rescued synaptic plasticity and cognitive function, and decreased AD pathology [31]	• National registry cohort studies show a reduced incidence of AD in people taking GLP analogues [25] • Secondary analysis of RCTs of GLP analogues focussing on cardio-vascular outcomes indicate reduced incidence of AD [25,32], • A pilot study of 38 patients with AD demonstrated significant benefits in glucose metabolism for liraglutide compared with placebo [33]. A phase 2b trial of liraglutide in mild-moderate AD indicated significant benefits in cognition and medial temporal atrophy [26]
Fasudil	· Reduction in Aβ plaque levels via the Dkk1-driven Wnt–PCP pathway [34] · Reduction of inflammation levels [35], rescue of synaptic damage [36], and reduced dendritic arborization [37]	· There is consistent evidence that fasudil protects synaptic function, reduces amyloid burden and rescues cognition in a range of animal models of AD [34,36,38],	· The safety and acceptability of this drug was shown in studies focussing on ischaemic heart disease [39] · One small study of people with MCI and AD suggested cognitive benefits with fasudil compared to nimodipine [40]
Phenserine	· Reduces apoptosis and impacts on cell death pathways [41] · Additional actions include suppression of IL-1β production [42], reduction in glutamate-induced excitotoxicity [43], protection against oxidative stress [43], and reduction in Aβ plaque levels [44] · Increase in production of brain-derived neurotrophic factor (BNDF) [43] · Inhibition of APP [44]	· Multiple pre-clinical studies demonstrated that phenserine alters cell death pathways, improves neuronal survival, and decreases APP levels in cell cultures and rodent models of AD, stroke, and head injury [[44], [45], [46]]	· A small RCT randomized 20 participants with mild AD to receive either phenserine (15 mg twice per day) or placebo for 3 months. The group of participants receiving phenserine had significantly better cognitive function at the 3-month time-point [47] · A phase II, 12-week RCT in 164 participants with AD indicated that (−)-phenserine (10–15 mg twice per day) had significantly better cognitive function than the group receiving placebo [48]
Herpes zoster vaccine	· Herpes zoster is thought to increase the risk of dementia by triggering neuroinflammation, generating oligomers, promoting the accumulation of amyloid plaques and neurofibrillary tangles of hyperphosphorylated tau, as well as causing vascular damage in the central nervous system and, in severe cases, encephalopathy [49]	The varicella zoster virus has recently been linked to amyloid deposition and aggregation of tau proteins [13], as well as cerebrovascular disease that resembles the patterns commonly seen in AD, such as small to large vessel disease, ischemia, infarction, and haemorrhage [14,18,19]. In addition, reactivations of the varicella zoster virus may in turn reactivate the herpes simplex virus in the brain [21]	Recent well-conducted epidemiological studies have consistently indicated a correlation between vaccination against herpes zoster, mostly using the active vaccine, have indicated a significant reduction in the incidence of dementia, including AD • Systematic review of 5 studies, with almost a million participants total, suggested a relative reduction of 16 % in incident dementia [50] • In subsequent studies, there was a 20 % relative reduction and 3.5 percentage point reduction in the probability of new dementia diagnoses compared with unvaccinated individuals • [51], with consistent findings from another recent report [52] • Most evidence is with the active vaccine, but a recent study did suggest a more modest reduction in dementia risk with the recombinant vaccine [53]
Riluzole	· Inhibition of glutamatergic neurotransmission and of voltage- dependent sodium channels [54] · Protection of neuronal firing from amyloid [55,56], and potentiation of postsynaptic GABA receptor function ((78) · Increase in BDNF levels [57] · Normalisation of EAAT3 expression [58,59], and of glucose metabolism [60] · Reduction of tau [61] and Aβ plaque burden [62] · Reduction of hippocampal AChE activity and of oxidative stress [63] · Reduction in levels of disease-associated microglia [62] · Increase in dendrite density [58]	Pre-clinical evidence in mice is in broad agreement that riluzole improves cognition in various mouse models of AD [64,[59], [60], [61], [62],^65^],**.**	One small 6-month clinical trial in 50 people with probable AD (MMSE, 19–27) reported that riluzole had a protective effect on brain glucose metabolism compared with placebo, with the most robust effect in posterior cingulate, and effects in precuneus, lateral temporal, right hippocampus, and frontal cortex [66]. Although underpowered for statistical evaluation, there were also numerical benefits on cognitive outcomes and a significant correlation between cognitive outcomes and PET biomarkers.
Sildenafil	· Increase in neurite growth [67] · Reduction in tau hyperphosphorylation [[67], [68], [69]] · Improvement in central nervous system hemodynamic function and increase in oxygen levels [70] · Reduction of hippocampal Aβ42 levels and in GFAP expression [71] · Reduction in α-synuclein levels and oxidative stress [72] · Rescue of PKG/pCREB signalling [73] · Decrease in GSK3β and CDK5 activity and increased BDNF and Arc levels [69] · Increase in levels of activated JNK (p-JNK) [74] and up-regulated heme oxygenase-1 [75] · Regulation of NO-cGMP signalling [76] · Down-regulation of pro-apoptotic proteins in aged mice [77]	· Pre-clinical evidence in mice is in broad agreement that riluzole improves cognition in various mouse models of AD [73,[78], [79], [80]],	· Epidemiological studies reporting contrasting findings on the protective effect of sildenafil on AD · One small open study of sildenafil in 8 patients with AD using a novel MRI technique to examine cerebral oxygen metabolism demonstrated a significant improvement in cerebral hemodynamic function with sildenafil treatment [70] · A further small MRI study in 10 patients with AD suggests that sildenafil appears to normalize spontaneous neural activity [81] No RCTs have been conducted

### 1.2 Identifying candidate combinations

It is highly likely that targeting different AD pathways may elicit combined independent benefits of the different treatment approaches, but in an ideal scenario, there would be additional synergistic benefits. The various pathological changes and pathways involved in AD are not independent but are intricately interconnected, forming a complex web of interactions that drives the progression of the disease pathology and related symptoms [82]. For instance, the accumulation of Aβ plaques and the formation of tau tangles can synergistically promote neuronal damage and cognitive decline [83]. Neuroinflammation can be triggered by the presence of Aβ plaques and tau, which in turn exacerbates their accumulation and contributes to neuronal loss [84]. Synaptic dysfunction can arise as a consequence of both Aβ and tau pathology, as well as the inflammatory milieu [85]. Furthermore, cerebrovascular dysfunction can impair the clearance of Aβ plaques, promote neuroinflammation, and induce oxidative stress, all of which contribute to neurodegenerative processes [86].

Beyond the core neuropathological hallmarks, cerebrovascular dysfunction also plays a significant role in the pathogenesis of AD [87]. Vascular risk factors such as hypertension, hyperlipidemia, and diabetes are associated with an increased risk of developing AD and can exacerbate its progression [88]. Cerebrovascular dysfunction can lead to reduced cerebral blood flow, hypoxia, and impaired clearance of Aβ plaques from the brain. This complex interplay underscores the necessity of developing combination therapies that use a smart approach to targeting AD pathways to achieve the maximum synergistic benefit on disease progression [20].

In order to identify potential combinations of high priority candidates as repurposed treatments for AD, we utilized two different approaches. Firstly, we focussed on the candidate therapies for repurposing identified by our Delphi panel from each of our three completed programmes. Eligible combinations focussed on one of the compounds identified as the highest priority candidates from any of the programmes, excluding compounds that have failed to confer benefit in subsequent RCTs, in combination with another high priority or shortlisted candidate. This limited the selection to treatments that were already identified as good candidate therapies. Combinations within this pool of compounds with the potential for synergies were then identified by the authors of the current paper through literature review, and the best combinations were prioritized by consensus by the author group. The nine highest priority combinations and the potential mechanistic synergies for these combinations are described in Table 2.

**Table 2:** Mechanistic synergies and rationales for therapy combinations arising from review of Delphi candidates.

Priority Repurposing Candidate	Candidate Combination Candidate	Mechanistic Synergy and Rationale
**Fasudil**	**Fingolimod**	Fasudil primarily targets the Rho-ROCK pathway to improve cerebrovascular function and inhibit microglial M1 activation. Fingolimod modulates adaptive immunity and intrinsic CNS inflammation by preventing peripheral immune cell entry. Fingolimod also supports synaptic health via brain-derived neurotrophic factor [[89], [90], [91], [92]]
	**Memantine**	Memantine preferentially blocks overactive extrasynaptic NMDA channels [92], protecting neurons from excitotoxicity. Fasudil improves synaptic plasticity and blood flow, creating a better environment for neurons [1]. Fasudil enhances the neuronal environment while memantine protects the neurons within it [89,92,93],
	**Vortioxetine**	Vortioxetine addresses neurotransmitter deficits and is a multimodal antidepressant. By antagonizing 5-HT₃ receptors, vortioxetine can disinhibit cholinergic and glutamatergic signalling to improve memory encoding [94]
	**Rifamycin**	Rifampicin disaggregates Aβ and tau oligomers in preclinical models. Fasudil inhibits RhoA/ROCK signalling to decrease tau phosphorylation. Rifampicin targets protein aggregates while fasudil improves tau clearance and cerebrovascular function [[95], [96], [97]]
**GLP-1**	**Microlithium**	GLP-1 analogues like liraglutide have shown neuroprotective effects in AD models and help maintain brain glucose metabolism. Lithium has disease-modifying properties and can be effective in mild cognitive impairment. The combination targets both metabolic dysfunction and tau pathology [[98], [99], [100]]
	**Phenserine**	GLP-1 analogues have shown neuroprotective effects by reducing amyloid and tau pathology. Phenserine is investigated as a potential disease-modifying therapy targeting APP. These agents target amyloid and tau pathology through distinct mechanisms [[101], [102], [103]]
**Sildenafil**	**SSRI (Vortioxetine / fluoxetine)**	Sildenafil increases cGMP levels, enhancing cerebral blood flow and synaptic plasticity, which can restore cognitive function in AD models. Chronic SSRI treatment can reduce Aβ deposition. These drugs enhance neuroplasticity and reduce AD pathology via distinct routes [69.^104^.^105^],
**Herpes zoster Vaccine**	**Anti-inflammatory**	Herpes zoster vaccination is associated with a lower risk of dementia [106], possibly by priming the immune system against viral triggers [107]. Long-term use of nonsteroidal anti-inflammatory drugs (NSAIDs) like ibuprofen has been associated with a reduced risk of AD [108]. An immune-priming effect from the vaccine could be complemented by the neuroinflammation-reducing effects of anti-inflammatory drugs

Secondly, we have taken a more data driven approach, examining the Clinical Practice Research Datalink (CPRD) primary care database in the UK to identify any treatment combinations associated with a reduced incidence of AD. We have previously reported an inverse association between individual drug prescriptions and AD incidence, based on a case-control analysis [109]. Building on this, we undertook a retrospective analysis of the same UK CPRD dataset of 40 million medical records to investigate potential synergistic effects between drug pairs in relation to AD susceptibility. Specifically, we evaluated whether combinations of two drugs, prescribed five to ten years prior to the index date, were associated with a modified risk of AD beyond the additive effects of each drug individually. To assess this, logistic regression models were fitted for each drug pair, including both main effects and their interaction term. The binary outcome variable indicated case/control status, and the predictors included indicators for exposure to each drug and their interaction. The evaluation specifically required a significant synergy score for the two identified therapies, as well as overall benefit, to increase the robustness of the analysis. The synergy score for a drug pair was defined as the z-value of the interaction term coefficient from this model. The significant synergistic pairings and their hypothetical synergistic mechanisms are shown in Table 3. Several drug combinations exhibited strong evidence of protective synergy. Notably, combinations such as beclometasone and salbutamol (*z* = –14.03) [[110], [111], [112]], clindamycin [113] and phosphate (*z* = –11.25) [114], and estradiol [115] and folic acid (*z* = –9.96) [116] showed greater protective effects than expected based on their individual associations. Many of the synergistic pairs involved hormonal therapies or anti-infective agents, consistent with emerging hypotheses linking endocrine and immune pathways to AD susceptibility. These findings highlight drug–drug interactions as a potentially important and underexplored factor in modulating AD risk. However, the exact biological mechanisms remain speculative, with further experimental validation needed as these associations only indicate correlation and not causation. It should also be acknowledged that there are many potential confounding factors with real-world datasets, and that the majority of candidates identified through this approach have not been shown to be effective therapies in further evaluations. Nevertheless, this approach is potentially valuable in informing further pre-clinical studies to verify potential mechanisms and benefits and to identify candidate combinations. More details and possible mechanisms for synergies in these combinations are shown in Table 3.

**Table 3:** *Potential protective synergistic effects between pairs of drugs prescribed 5 to 10 years prior to AD diagnosis.* Logistic regression–derived odds ratios (ORs) and 95 % confidence intervals are reported for each drug individually and for their combined use. The strength and significance of the interaction (ie, departure from additivity on the log-odds scale) is indicated by the z-score of the interaction term from the regression model. The hypothetical synergistic action is summarised.

	Drug A	Drug B	Synergy Hypothesis
**Candidate**	**Beclometasone**	**Salbutamol**	**Anti-inflammatory:** Chronic systemic inflammation is a significant risk factor for AD. Beclometasone's potent anti-inflammatory action could reduce neuroinflammation [110]. Salbutamol, while primarily a bronchodilator, has anti-inflammatory properties [111], potentially by modulating immune cell activity or cytokine release [112]. **Beyond Respiratory:** β2 adrenergic receptors are present in the brain. Modulation by salbutamol might have direct neuroprotective effects, especially when combined with the anti-inflammatory action of beclometasone
**OR** **(95 % CI)**	−0.02 (−0.04 0.00)	−0.17 (0.20–0.15)	
	−0.23 (−0.26 − 0.20)		
**Synergy Z Score**	−14.03		
**Candidate**	**Clindamycin**	**Phosphate**	**Microbiome Modulation:** Clindamycin is a broad-spectrum antibiotic, significantly altering gut microbiome [113]. The gut-brain axis is increasingly recognized as a key player in AD [117]. Dysbiosis can lead to increased gut permeability, systemic inflammation, and production of neurotoxic metabolites. **Cellular Energy Metabolism:** Phosphate is crucial for numerous cellular processes, including ATP production, DNA synthesis, and cell signalling [114]. While excess phosphate can be detrimental, its combination with clindamycin could suggest a complex interplay. Clindamycin's microbiome modulation could make cells more efficient at utilizing phosphate for energy or repair, or phosphate may act as a cofactor in specific metabolic pathways that are protective against AD when the microbiome is altered by clindamycin **Inflammation/Oxidative Stress:** A healthy gut microbiome influences systemic inflammation and oxidative stress [113]
**OR** **(95 % CI)**	−1.48 (−1.57–1.38)	0.23 (0.20 0.25)	
	1.74 (−1.87–1.62)		
**Synergy Z Score**	−11.25		
**Candidate**	**Estradiol**	**Folic Acid**	**Hormonal Neuroprotection:** Estradiol has neuroprotective effects [115], including antioxidant properties, promotion of neuronal survival, modulation of neurotransmitter systems, and maintenance of cerebral blood flow [[117], [118], [119]] **Homocysteine Reduction and Vascular Health:** Folic acid is essential for homocysteine metabolism, a risk factor for cardiovascular disease and AD. By reducing homocysteine levels, folic acid improves brain vascular health, reducing the risk of cerebrovascular pathology that contributes to AD [116] **Epigenetic:** Folate is involved in methylation, which is crucial for epigenetic regulation. Both estrogen and folate influence epigenetic modifications **Synaptic Plasticity and Neurogenesis:** Both estrogen and folate have been implicated in promoting synaptic plasticity and neurogenesis. Their combined action could lead to enhanced cognitive reserve and resilience against AD pathology
**OR** **(95 % CI)**	−1.51 (−1.55 − 4.48)	−0.22 (−0.27–0.18)	
	2.33 (−2.52 − 2.15)		
**Synergy Z Score**	−9.96		

### 1.3 Some caution regarding combination approaches

While combining therapies that target multiple disease pathways offers clear theoretical advantages, this approach must be considered against the backdrop of risks associated with current and future treatments, particularly in vulnerable populations affected by AD. For instance, the recent advent of anti-amyloid monoclonal antibodies has highlighted specific safety concerns such as Amyloid-Related Imaging Abnormalities (ARIA), which can manifest as cerebral edema or microhemorrhages [120]. These risks are especially pertinent in the target population of older adults, many with Mild Cognitive Impairment (MCI) who are already at an increased baseline risk for falls, confusion, and cognitive fluctuations. In the broader group of individuals with AD or other dementias, the majority of people are over 80, many presenting with multiple comorbidities such as cardiovascular and renal disease, necessitating numerous concomitant medications [121]. Introducing a combination therapy into such a complex polypharmacy regimen significantly increases the potential for adverse drug-drug interactions and cumulative tolerability issues. These patient-centric challenges are mirrored by methodological hurdles, including the increased complexity and cost of clinical trial designs and the additional regulatory scrutiny required when evaluating two or more active treatments to demonstrate the benefits of each agent as well as the combination [122]. Therefore, novel strategies such as network pharmacology and biomarker-guided precision medicine will be key to developing and optimizing patient-specific regimens that maximize benefit while minimizing harm [13,123]. Fluid biomarkers in AD offer a minimally invasive means to detect and monitor underlying pathological changes, including amyloid-beta accumulation, tau pathology, and neurodegeneration. Key CSF biomarkers include decreased Aβ42 (reflecting plaque deposition) and increased p-tau and total tau, which correlate with tangle pathology and neuronal injury. Recent advances have enabled the development of blood-based biomarkers, such as plasma Aβ42/40 ratio, p-tau181, p-tau217, and Nfl, which closely mirror CSF and PET findings and are increasingly used in clinical trials and early diagnostic frameworks [124]. These biomarkers facilitate early diagnosis, patient stratification, and treatment monitoring, and are central to the move toward precision medicine in AD. A related article in this special issue explores the integration of biomarkers in combination therapy (*Author list TBD*, 2025).

## 2 Conclusions and recommendations

Although it is important to be mindful of potential design, regulatory, and tolerability issues with combination therapies, the likely benefits should outweigh the risks for carefully selected, synergistic treatments. Using two different approaches—expert review and evaluation of a large real-world dataset—we have identified 17 potential combinations to inform further pre-clinical and clinical studies.

From a pragmatic perspective, low-risk treatment combinations are well suited to large-scale hybrid trial designs run substantively through digital platforms. This will have the advantage of cost-effective, well-powered trials with the power to detect the additive effect of treatment combinations as a critical proof of concept of the combination treatment approach. Although the current paper focuses on pharmacological treatment combinations, it will also be important to consider the potential additional benefits of combining treatments with lifestyle interventions to maximise benefit.

## CRediT authorship contribution statement

**Clive Ballard:** Writing – review & editing, Writing – original draft, Methodology, Formal analysis, Data curation, Conceptualization. **Janet Sultana:** Writing – review & editing, Writing – original draft, Formal analysis, Data curation. **Pat Doherty:** Writing – review & editing, Writing – original draft, Formal analysis, Data curation. **Gareth Williams:** Writing – review & editing, Writing – original draft, Formal analysis, Data curation. **Anne Corbett:** Writing – review & editing, Writing – original draft, Formal analysis, Data curation.

## Declaration of competing interest

The authors declare the following financial interests/personal relationships which may be considered as potential competing interests: Anne Corbett reports a relationship with Addex Therapeutics Ltd that includes: consulting or advisory. Anne Corbett reports a relationship with Acadia Pharmaceuticals Inc that includes: consulting or advisory. Suven reports a relationship with Janssen Pharmaceuticals Inc that includes: consulting or advisory. Anne Corbett reports a relationship with Novo Nordisk that includes: funding grants. Anne Corbett reports a relationship with Therini Bio that includes: funding grants. Anne Corbett reports a relationship with reMYND Nv that includes: funding grants. Anne Corbett reports a relationship with Synexus Clinical Research Ltd that includes: funding grants. Clive Ballard reports a relationship with Acadia Pharmaceuticals Inc that includes: consulting or advisory. Clive Ballard reports a relationship with AARP that includes: consulting or advisory. Clive Ballard reports a relationship with Addex Therapeutics Ltd that includes: consulting or advisory. Clive Ballard reports a relationship with Eli Lilly that includes: consulting or advisory. Clive Ballard reports a relationship with Enterin Inc that includes: consulting or advisory. Clive Ballard reports a relationship with GWPharm that includes: consulting or advisory. Clive Ballard reports a relationship with Lundbeck LLC that includes: consulting or advisory. Clive Ballard reports a relationship with Novartis that includes: consulting or advisory and funding grants. Clive Ballard reports a relationship with Janssen Pharmaceuticals Inc that includes: consulting or advisory. Clive Ballard reports a relationship with Johnson & Johnson MedTech that includes: consulting or advisory. Clive Ballard reports a relationship with Novo Nordisk that includes: consulting or advisory and funding grants. Clive Ballard reports a relationship with Otsuka Pharmaceutical Co Ltd that includes: consulting or advisory. Clive Ballard reports a relationship with Suven Life Sciences Limited that includes: consulting or advisory. Clive Ballard reports a relationship with Synexus Clinical Research Ltd that includes: consulting or advisory and funding grants. Clive Ballard reports a relationship with tauX that includes: consulting or advisory. If there are other authors, they declare that they have no known competing financial interests or personal relationships that could have appeared to influence the work reported in this paper.
